# Sex-specific mouse liver gene expression: genome-wide analysis of developmental changes from pre-pubertal period to young adulthood

**DOI:** 10.1186/2042-6410-3-9

**Published:** 2012-04-04

**Authors:** Tara L Conforto, David J Waxman

**Affiliations:** 1 Division of Cell and Molecular Biology, Department of Biology, Boston University, Boston, MA 02215, USA

**Keywords:** Pre-pubertal development, Liver gene expression, Sexual dimorphism, Microarray analysis, Growth hormone

## Abstract

**Background:**

Early liver development and the transcriptional transitions during hepatogenesis are well characterized. However, gene expression changes during the late postnatal/pre-pubertal to young adulthood period are less well understood, especially with regards to sex-specific gene expression.

**Methods:**

Microarray analysis of male and female mouse liver was carried out at 3, 4, and 8 wk of age to elucidate developmental changes in gene expression from the late postnatal/pre-pubertal period to young adulthood.

**Results:**

A large number of sex-biased and sex-independent genes showed significant changes during this developmental period. Notably, sex-independent genes involved in cell cycle, chromosome condensation, and DNA replication were down regulated from 3 wk to 8 wk, while genes associated with metal ion binding, ion transport and kinase activity were up regulated. A majority of genes showing sex differential expression in adult liver did not display sex differences prior to puberty, at which time extensive changes in sex-specific gene expression were seen, primarily in males. Thus, in male liver, 76% of male-specific genes were up regulated and 47% of female-specific genes were down regulated from 3 to 8 wk of age, whereas in female liver 67% of sex-specific genes showed no significant change in expression. In both sexes, genes up regulated from 3 to 8 wk were significantly enriched (*p *< E-76) in the set of genes positively regulated by the liver transcription factor HNF4α, as determined in a liver-specific HNF4α knockout mouse model, while genes down regulated during this developmental period showed significant enrichment (*p *< E-65) for negative regulation by HNF4α. Significant enrichment of the developmentally regulated genes in the set of genes subject to positive and negative regulation by pituitary hormone was also observed. Five sex-specific transcriptional regulators showed sex-specific expression at 4 wk (male-specific *Ihh; *female-specific *Cdx4, Cux2, Tox*, and *Trim24*) and may contribute to the developmental changes that lead to global acquisition of liver sex-specificity by 8 wk of age.

**Conclusions:**

Overall, the observed changes in gene expression during postnatal liver development reflect the deceleration of liver growth and the induction of specialized liver functions, with widespread changes in sex-specific gene expression primarily occurring in male liver.

## Background

The liver performs a variety of physiological functions including glycogen storage, cholesterol catabolism to bile acids, and drug metabolism [1,2]. Liver development begins around embryonic day 9 (E9) in the mouse and the transcriptional transitions during hepatogenesis are well characterized [1]. Changes in gene expression during the postnatal/pre-pubertal period are less well understood [3], especially with regards to sex-biased gene expression. Over 1,000 genes are known to be differentially expressed between male and female liver and are regulated primarily by pituitary patterns of growth hormone secretion [4-6], which are sex-dependent and subject to regulation by estrogen and testosterone [7].

During early mouse postnatal development, rapid growth of the body and somatic organs occurs and then slows down as the animal ages. From 2 to 4 wk of age liver mass increase is predominantly due to polyploidization and to a lesser extent, hypertrophy [8], while from 4 to 8 wk there is a decrease in polyploidzation and the increase in liver mass is primarily due to hyperplasia. *β-catenin*, a gene associated with cell proliferation, is induced at postnatal day 5 and highly expressed until postnatal day 20 [9]. Little growth of the liver is observed after 8 wk, at which time growth-promoting genes active at 1 wk, such as *Igf2, Mest, Peg3*, are repressed as they are in other tissues showing decelerated growth, including kidney, lung, and heart [10]. There is a reciprocal relationship between the decrease in *Igf2 *expression and the increase of *Igf1 *expression during postnatal mouse liver development [11]. The developmental decreases in somatic growth and in the expression of *Igf2, Mest, Peg3 *appear to be regulated by the size and not the age of the animal *per se*, as seen in studies where the anti-thyroid drug propylthiouracil is used to inhibit body growth [10].

Large changes in gene expression occur in early postnatal mouse liver as the liver's primary function shifts from hematopoiesis to specialized liver functions [3]. RNA polymerase activity steadily increases and plateaus at 30 days of age [12], while RNA synthesis peaks at 60 days and decreases at later ages. Innate immune system genes are activated from E18.5 to postnatal day 3, while liver function genes, such as those involved in lipid and fatty acid metabolism, are activated at postnatal day 7 [3]. In rat liver, changes in expression of genes associated with metabolism occur around the time of weaning [13]. In particular, gluconeogenesis and ketogenic enzyme activity decrease, while glucokinase and lipogenic activity increase [13]. A decrease in respiration rates occurs in mouse liver mitochondria right after weaning, but the levels later recover in adults [14]. These changes in metabolic enzyme activity may be due to a rise in thyroid hormone and/or glucocorticoid levels [13].

Genes encoding cytochromes P450 and other enzymes of drug and steroid metabolism show marked changes in expression during postnatal liver development. In male and female mouse liver, *Cyp1a2, Cyp2d33, Cyp2f2, Cyp3a13*, and *Cyp3a25 *are expressed at low levels at postnatal day 10 or 15 and then increase until day 20, after which the expression is largely maintained [15]. A similar pattern of expression was observed for *CYP1A2 *and *CYP2E1 *in male rat liver [16]. Many *Cyps *and other genes active in drug metabolism show sex-differences in expression beginning at puberty [6,15,17-19]. Genes such as *Cyp2b9, Cyp3a41*, and *Cyp3a44*, are expressed at high levels in both male and female mouse liver prior to puberty, but are then selectively repressed in male liver, resulting in female-biased expression at adulthood [18-20]. In the rat, the male-specific *CYP2C11 *and the female-specific *CYP2C12 *are both expressed at low levels in both male and female liver until puberty, at which time *CYP2C11 *is up regulated in male liver and *CYP2C12 *is up regulated in female [21,22]. *Sult *genes involved in hydroxysteroid sulfate conjugation display sex-specific expression after puberty in mouse liver [17,23]. Six of seven *Sult2a *genes show high pre-pubertal expression in both male and female liver, however in adult mouse liver, three *Sult2a *genes display female-specific expression while three others decrease in expression in both male and female liver [17]. The seventh *Sult2a *gene is not detectable prior to puberty and is expressed at adulthood in a sex-independent manner [17].

Presently, we investigate on a global scale the effects of age and sex on gene expression in mouse liver. We compare gene expression profiles in the pre-pubertal period (3 wk and 4 wk of age) to the post-pubertal/young adult stage (8 wk old) and find that changes in sex-specific gene expression primarily occur in male liver. We also show that genes that show changes during this period of development are enriched in the set of genes whose expression is dependent on the liver transcription factor hepatocyte nuclear factor 4α (HNF4α) [24], as determined using a liver-specific mouse HNF4α knockout model [25]. Finally, we identify other transcription factors that show significant changes in expression during postnatal development and may contribute to the observed changes in liver gene transcription during this developmental period.

## Methods

### Animal treatments, liver RNA isolation, and quantitative PCR (qPCR)

Surrogate mothers with 7 day old male and female crl:CD1 mouse pups were purchased from Charles River Labs (Wilmington, MA). CD1 mice were chosen for this study due to the extensive earlier mechanistic studies of sex-specific liver gene expression carried out in this strain; these include global analysis of DNase I hypersensitivity, responsiveness to hypophysectomy and growth hormone treatment, and the identification of binding sites for the growth hormone-regulated transcription factors STAT5 and BCL6 [26-28]. Male and female mice were killed at 3, 4, or 8 wk of age (n = 10-12 mice/sex/age group) and livers were snap frozen in liquid nitrogen and stored at -80°C. Total RNA was isolated from frozen individual livers using TRIzol reagent (Invitrogen, Carlsbad, CA). RNA samples were converted to cDNA using a high-capacity cDNA reverse transcription kit (Applied Biosystems, Foster City, CA). Triplicate 5-μl real-time PCR mixtures, each containing Power SYBR green PCR master mix (Applied Biosystems), 312 nM each qPCR primer, and 0.5-1.5 μl DNA template were loaded onto a 384-well plate and run through 40 cycles on an ABS 7900HT sequence detection system (Applied Biosystems). Data were graphed as relative values, normalized to the 18S rRNA content of each sample. Statistical analyses were carried out by 2-way ANOVA using PRISM software version 4 (GraphPad, Inc., San Diego, CA). qPCR primers are listed in Additional file 1. RNA integrity (minimum RIN number 8.0) was verified using an Agilent Bioanalyzer 2100 instrument (Agilent Technology, Palo Alto CA).

### Microarray analysis

Seven independent competitive hybridization microarray experiments were carried out: 1) Male 3 wk vs. Female 3 wk; 2) Male 4 wk vs. Female 4 wk; 3) Male 8 wk vs. Female 8 wk; 4) Male 3 wk vs. Male 8 wk; 5) Male 4 wk vs. Male 8 wk; 6) Female 3 wk vs. Female 8 wk; and 7) Female 4 wk vs. Female 8 wk. Two independent pairs of pooled liver RNA randomized samples were used for each microarray comparison; these biological replicates were analyzed as dye swaps to correct for dye bias, giving a total of 14 microarrays. To minimize the impact of individual mouse to mouse variability on the microarray data, each biological replicate was comprised of a randomized pool of liver RNA prepared from n = 5-6 mice. Thus, for array comparison 1, Male 3 wk liver pool A was labeled with Alexa 555 dye and Female 3 wk liver pool A was labeled with Alexa 647 dye (biological replicate one), and Male 3 wk liver pool B was labeled with Alexa 647 dye and Female 3 wk liver pool B was labeled with Alexa 555 dye (biological replicate two); array comparison 1 is thus based on comparisons of 10-12 individual male mice and 10-12 individual female mice at each age. Hybridization of fluorescent labeled RNA to 39,429-feature Agilent microarrays was carried out for each pair of independent biological replicates, giving a total of 14 arrays across the 7 array comparisons. SurePrint G3 4×44 K Mouse Gene Expression microarrays (catalog no. G4846A-026655; Agilent Technology; Gene expression omnibus (GEO) platforms GPL10333 and GPL11202) were used for these studies.

Linear and LOWESS normalization were performed for each microarray using Agilent Feature Extraction software. The Feature Extraction analysis also calculates the variation of pixel intensity for each feature (spot) on the array. These error measurements were input to the Rosetta error model, which was used for subsequent analysis of statistical significance of differential gene expression. The Rosetta error model provides a gene-specific estimate of error by incorporating two elements: a technology-specific estimate of error and an error estimate derived from replicate arrays [29]. The technology-specific component utilizes an intensity-dependent model of error derived from numerous self-self hybridizations. In this study, two arrays, based on independent pools of biological replicates, were used for each comparison of interest. By including the technology-specific estimate, the Rosetta error model is able to avoid false positives that occur from under-estimation of error when a small number of replicate arrays are available, thus resulting in an increase in statistical power equivalent to that which would be obtained with at least one additional replicate. For two-color microarrays a log-ratio error estimate is derived in the Rosetta error model from the individual error estimates of each sample (color) used in the co-hybridization. Then, for each feature an average log ratio and associated *p*-value are obtained from replicate measurements (arrays) using the Rosetta error model error-weighted averaging method. In this approach, the average ratio is calculated by weighting the ratio from each sample inversely proportional to the variance of that sample. This produces an averaged ratio with the smallest possible error. Validation with spike-in experiments has demonstrated that the Rosetta error model has superior accuracy in detecting and quantifying relative gene expression when compared to other statistical methods commonly used in microarray analysis [30]. Direct experimental validation is provided in the present study, where qPCR analysis of n = 9-10 individuals per group gave results indistinguishable from those obtained by microarray analysis (see below).

The statistical significance of differential expression of each gene was determined by application of a filter (*p *< 0.0001) to the Rosetta *p*-values. All together, 7,915 probes met this *p*-value threshold in one or more of the 7 array comparisons, after removal of redundant probes (see below) and after removal of 142 probes that did not pass Agilent's "well above background" condition, which requires a probe's raw signal to be greater than 99% of the background population signal. The number of microarray probes expected to meet the significance threshold of *p *< 0.0001 by chance is 0.0001 × 39,429 probes or 4 probes. The actual number of probes that met this *p*-value ranged from 104 (Male 3 wk vs. Female 3 wk arrays), to 3,784 (Male 3 wk vs. Male 8 wk arrays) across the seven array comparisons, corresponding to a false discovery rate ranging from 4/104 (3.85%) to 4/3,784 (0.11%). Further, a |fold-change| filter of > 1.5-fold was combined with the *p *< 0.0001 filter to limit consideration to genes showing expression ratios > 1.5 (up regulated genes) or < 0.667 (down regulated genes). When two or more probes assigned the same gene name gave the same pattern of regulation across the seven microarray comparisons, they were deemed to be redundant probes (as indicated by assignment to the same total flag sum (TFS) group; see Additional file 2 and below), and only the probe with the lowest set of *p*-values was retained. Probes associated with the same gene name but different TFS groups were retained. After removing redundant probes, 5,715 probes (genes) met the above specified *p*-value, expression ratio, and well above background filters. 1,212 of the 5,715 genes showed significant sex-differences in expression at 8 wk, and on that basis were defined as adult sex-specific (adult sex-biased) genes. All microarray data files are available at the GEO web site [31] as GEO series GSE34782.

### Data analysis

A system of binary and decimal flags (TFS) [32] was used to classify the 5,715 regulated genes based on expression ratios and *p*-values across the seven microarray comparisons (Additional file 2). Hierarchical clustering and heat map generation were carried out separately for the 1,212 adult sex-specific genes and 4,503 sex-independent genes using Cluster [33] and Java TreeView [34]. STEM clustering [35,36] of the adult sex-specific genes, and separately, STEM clustering of the adult sex-independent genes, was used to identify common patterns present within each gene set. STEM clustering was carried out using log2 gene expression ratios from our microarray study, with each of the 7 microarrays serving as a separate data point for STEM clustering. The maximum number of model STEM profiles considered was set to either 30 or 50 and the maximum unit change in model profiles between time points was set equal to 2 [35,36]. The STEM profiles are derived from predefined patterns based on the maximum number of profiles and the maximum distance between two data points, which is selected by the user. A greedy approximation algorithm generates a set of patterns that maximizes the minimum distance between any two patterns so that the set of patterns is distinctive but also representative of all the possible patterns [35,36]. A permutation-based test was used to quantify the expected number of genes that would be assigned to each predefined pattern if the data were randomly generated. If the number of genes assigned to a given predefined pattern is significantly greater than the predicted number of genes, then the profile with the genes assigned to that predefined pattern is assigned a significant *p*-value.

The DAVID annotation tool [37,38] was used to analyze the genes in each STEM cluster to identify enrichment clusters deemed significant (minimum enrichment score of 1.3, which is equivalent to a *p*-value of 0.05). Potential transcriptional regulators were identified by searching the Gene Ontology (GO) descriptors of the 5,715 regulated genes for the terms "DNA binding" or "transcription". The initial list of genes was filtered using the following more stringent criteria: microarray signal intensity ≥ 25 at 8 wk of age, expression ratio > 2-fold in the arrays in which the gene exhibited a *p*-value < 0.0001, and in the case of adult sex-biased transcriptional regulators, expression ratio > 2 (or < 0.5) for the male 8 wk vs. female 8 wk comparison. The list of adult sex-specific transcriptional regulators were further narrowed down by focusing on genes whose expression was altered by conditions that influence the expression of adult sex-specific genes, such as hypophysectomy [27], STAT5b deficiency [32], and HNF4α deficiency [24,39].

### Comparison of microarray data sets and enrichment score calculations

Gene sets identified by microarray analysis of hypophysectomized mouse liver [27] or HNF4α-deficient mouse liver [24] were compared to the sets of genes that showed significant changes in expression from 3 wk to 8 wk in the present study. For these analyses, a more stringent threshold was used to define sex-independent genes, to exclude genes showing a weak sex-bias in expression as well as genes giving very low microarray signal intensities. Thus, the stringent criteria used to identify sex-independent genes was male:female (or female:male) expression ratio < 1.2, *p*-value > 0.01, and Agilent microarray signal intensity ≥ 25. Enrichment scores calculated for comparisons between microarray studies run on different array platforms were based on to the genes represented of both microarray platforms (Agilent mouse microarray G4122F-014868 used previously [24,27] vs. Agilent mouse microarray G4846A-026655 for the present study). A background gene set comprised of all genes common to both platforms was used when calculating enrichment scores for male-specific, female-specific, and all genes that are either up or down regulated from 3 wk to 8 wk. A background gene set comprised of all sex-independent genes was used as the background when calculating enrichment scores for sex-independent genes that are either up or down regulated from 3 wk to 8 wk. Enrichment *p*-values were calculated using two tail Fisher Exact Test, with a *p*-value < 0.0001 deemed significant.

## Results

### Overall patterns of developmental change in sex-independent mouse liver genes

Expression microarrays were used to investigate global changes in gene expression in male and female mouse liver from the pre-pubertal period (3-4 wk) to young adulthood (8 wk of age). Liver RNA was isolated from groups of male and female mice euthanized at each developmental time point and analyzed in seven sets of competitive microarray hybridization experiments (see Additional file 3A). Normalized expression ratios and *p*-values were calculated for all seven datasets using Rosetta Resolver software. 5,715 microarray probes (genes) met the combined threshold criteria for differential expression (expression ratio > 1.5 or < 0.667, and *p *< 0.0001) for at least one of the seven arrays after elimination of redundant probes (Additional file 3B). Genes were classified as male-specific (587 genes), female-specific (625 genes), or sex-independent (4,503 genes) based on their expression profiles in male compared to female liver at 8 wk of age (Table 1).

**Table 1:** Gene count distribution and onset of sex specificity

Sex specificity (defined at 8 wk)	Onset at 3 wk	Onset at 4 wk	Onset at 8 wk	No sex specificity	Total genes
		**Number of genes**			

Male-specific	7	54	526*	0	587

Female-specific	9	104	512**	0	625

Sex-independent	23 (M), 56 (F)***	59 (M), 86 (F)***	0	4279	4503

The onset of sex-specificity for 1,037 out of 1,212 adult sex-specific genes occurred after 4 wk and by 8 wk. 224 of the 4,503 adult sex-independent genes met the thresholds for sex-differential expression at 3 or 4 wk, but not at 8 wk, however, only 13 of these genes showed the same sex-bias at 3 wk as at 4 wk. Three of the 7 male-specific genes showing sex specificity at 3 wk are Y-chromosome genes

* Includes 5 genes found to be male-specific at 8 wk, but female-specific at 3 or 4 wk

** Includes 6 genes found to female-specific at 8 wk, but male-specific at 3 or 4 wk

*** These genes exhibit either male-specificity (M) or female-specificity (F) at 3 or 4 wk, as noted, but show sex-independent expression at 8 wk

**Figure 1:**
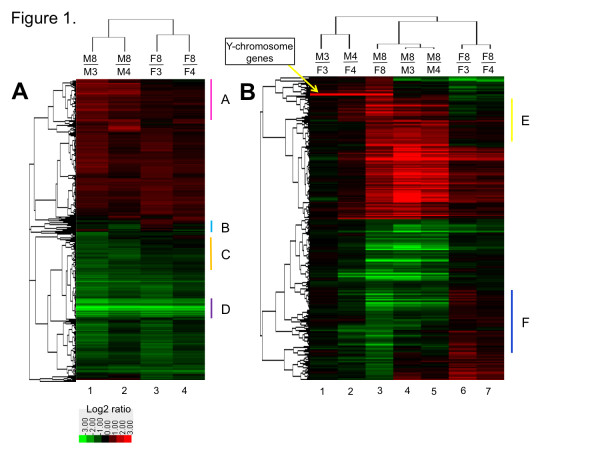
**Heat maps displaying patterns of expression for 5,715 regulated genes clustered by gene and sample**. Genes are depicted based on their expression ratios across the seven microarray experiments. Colors range from bright red (up regulation, log2 ratio ≥ 3) to bright green (down regulation, log2 ratio ≤ -3). Hierarchical clustering was performed based on Pearson's correlation of log2 ratios. The dendograms at the top identify arrays showing the greatest similarity in their patterns of expression. M3, male liver at 3 wk; M4, male liver at 4 wk; M8, male liver at 8 wk; F3, female liver at 3 wk; F4, female liver at 4 wk; and F8, female liver at 8 wk. **A**. Heat map showing expression ratios for 4,503 adult sex-independent genes in the four microarray comparisons indicated at the top. Qualitative differences between sexes were seen in the developmental changes for the gene groups marked A-C at the right: group A genes showed greater up regulation in male liver than in female liver, group B genes showed greater up regulation in female liver than in male liver, and group C genes showed greater down regulation in male liver than in female liver. Group D genes showed strong down regulation in both sexes from 3 or 4 wk to 8 wk and includes genes associated with cell cycle and mitosis. **B**. Heat map showing expression ratios for 1,212 adult sex-specific genes across the seven microarray comparisons indicated at the top. Marked at the right are genes in group E, which show developmental up regulation from 3 or 4 wk to 8 wk in male liver but not in female liver, and genes in group F, which show developmental up regulation in female liver and down regulation in male liver.

Hierarchical clustering of the 4,503 genes showing sex-independent expression at 8 wk showed that arrays from the same sex clustered together (Figure 1A). Many of the genes that were either up regulated (red) or down regulated (green) from 3 or 4 wk to 8 wk displayed similar changes in expression in both sexes. However, qualitative differences in developmental patterns between male and female liver were apparent (Figure 1A, gene groups marked A-C). Strong down regulation was seen in both sexes for some adult sex-independent genes (Figure 1A, group D), including genes involved in mitosis and the cell cycle, which reflects the decrease in cell growth that occurs in liver during the post-pubertal period [10].

### Developmental regulation of sex-biased genes

**Figure 2:**
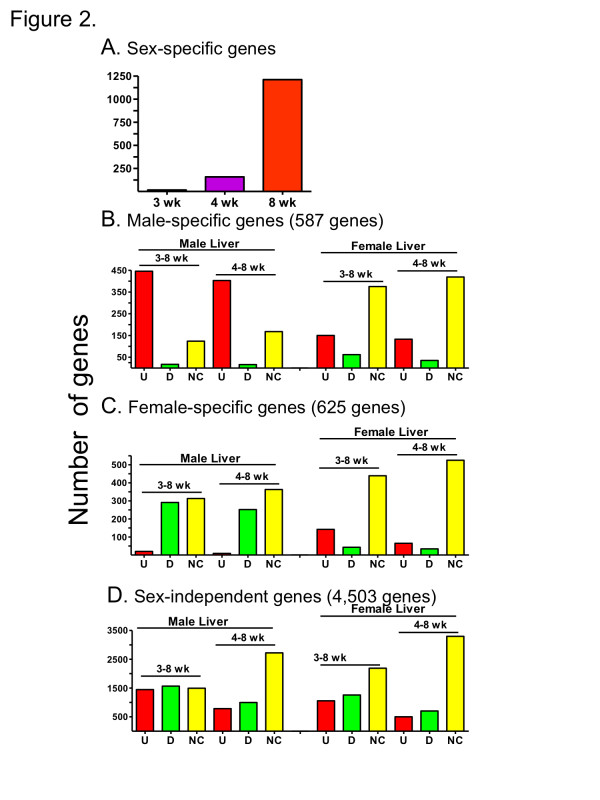
**Numbers of sex-specific genes and patterns of developmental regulation of adult sex-specific genes and adult sex-independent genes**. Bar graphs present the number of genes that are either sex-specific in adult mouse liver or are up regulated (U), down regulated (D), or not significantly changed in expression (NC) from 3 wk to 8 wk or from 4 wk to 8 wk in male and female liver, as marked. **A**. The number of adult sex-specific genes increases from 3 to 8 wk, with onset of sex-specificity primarily occurring between 4 wk and 8 wk. **B**. Adult male-specific genes are predominantly up regulated in male liver, while in female liver they are mostly not regulated. **C**. Adult female-specific genes are primarily down regulated or are not regulated in male liver, while in female liver they are mostly not regulated. **D**. A somewhat greater number of adult sex-independent genes are regulated from 3 or 4 wk to 8 wk in male liver compared to female liver.

Hierarchical clustering of the 1,212 adult sex-specific genes revealed similar numbers of male-biased genes as female-biased genes (Figure 1B, array 3). Sex differences in gene expression primarily emerged after 4 wk of age, with very few adult sex-specific genes showing sex-biased expression at 3 wk or 4 wk (Figure 1B, arrays 1 and 2 vs. array 3; Figure 2A). Thus, only 16 of the adult sex-specific genes showed sex-differential expression at 3 wk (Table 1). This increased to 174 genes by 4 wk, with two thirds of these genes showing female specificity. However, for the majority (86%) of adult sex-specific genes, sex-specific expression was first observed at 8 wk (Table 1, Figure 2A).

Approximately 1,000 genes showed a change in expression from 3 or 4 wk to 8 wk in male liver as compared to female liver (Additional file 3A). The largest number of genes showed postnatal developmental changes in either male liver only (2,348 genes) or in both male and female liver in a common manner (1,926 genes), as compared to changes in female liver only (981 genes) (Additional file 3C). In particular, many more adult sex-biased genes showed post-pubertal developmental changes in male liver as compared to female liver (Figure 1B, arrays 4 and 5 vs. arrays 6 and 7; Figure 2B, C). Thus, 488 (83%) male-specific genes showed a developmental change in male liver vs. 248 (42%) showed such a change in female liver. Similarly, 358 (57%) of female-specific genes showed a developmental change in male liver vs. 208 (33%) showed such a change in female liver (Additional file 3C). Moreover, 516 (43%) of the adult sex-biased genes displayed a developmental change in male liver only, while 126 (10%) showed a change in expression in female liver only (Additional file 3C). This greater frequency of developmental changes in male liver was seen for both male-specific and female-specific genes: 273 of 587 male-specific genes showed a developmental change in male liver only, while only 33 were changed in female only. Similarly, 243 of 625 female-specific genes were changed in expression in male liver only, while only 93 showed a female-specific change (Additional file 3C).

In male liver, almost all male-biased genes showing a developmental change were up regulated, while almost half of female-biased genes were down regulated after puberty. In contrast, in female liver, the majority of sex-biased genes showed no significant change in expression over the same period (Figure 2B, C). Fewer developmental changes in sex-independent genes were also seen in female liver (Figure 2D). Differences in the direction of regulation between male and female liver were noted for some genes. Examples include genes up regulated in male liver after 3-4 wk but unchanged in female liver, and genes down regulated in male liver but unchanged or slightly up regulated in female liver (Figure 1B, groups E and F, respectively).

### Clustering by predefined patterns (STEM analysis)

**Figure 3:**
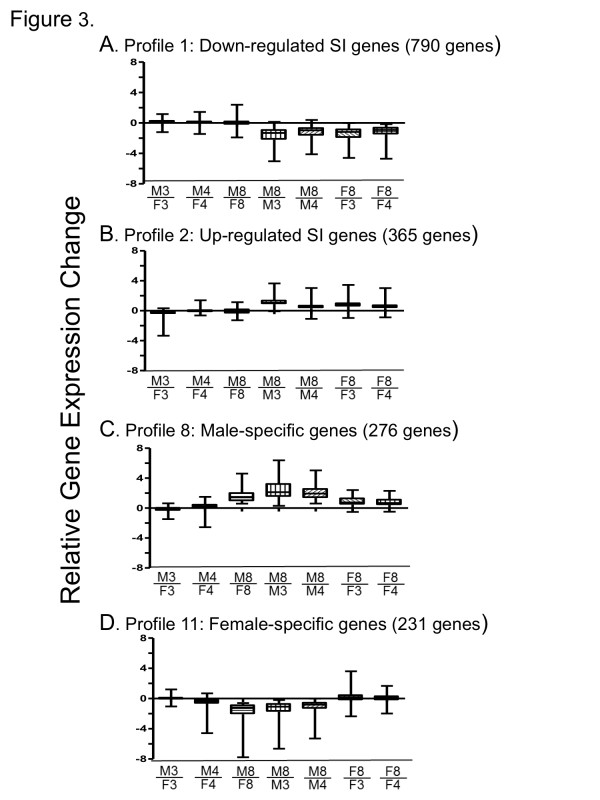
**Box and whisker plots representing gene expression patterns in genes clustered by STEM profiles**. Boxes represent the 25^th ^to the 75^th ^percentile of gene expression ratios for each of the 7 microarray comparisons indicated below the X-axis. A horizontal line across each box indicates the median expression ratio. The whiskers that extend above and below each box represent the highest and the lowest values. M3, male liver at 3 wk; M4, male liver at 4 wk; M8, male liver at 8 wk; F3, female liver at 3 wk; F4, female liver at 4 wk; and F8, female liver at 8 wk. **A**. Profile 1 is the largest adult sex-independent (SI) STEM cluster comprised of down regulated genes. On average, these genes are slightly more down regulated from 3 to 8 wk than from 4 to 8 wk in both male and female liver. **B**. Profile 2 is the largest adult sex-independent STEM cluster comprised of up regulated genes. These genes are slightly more up regulated from 3 to 8 wk than from 4 to 8 wk in both male and female liver. **C**. Profile 8 is the largest adult male-specific STEM cluster. These genes are up regulated from 3 to 8 wk and from 4 to 8 wk in male liver and to a lesser extent in female liver. **D**. Profile 11 is the large adult female-specific STEM cluster. These genes are down regulated from 3 to 8 wk and from 4 to 8 wk in male but not female liver.

The 5,715 genes that met our threshold criteria for at least one of the seven microarray comparisons were clustered using Short Time-series Expression Miner (STEM) [35]. STEM analysis was carried out separately for adult sex-specific and adult sex-independent genes; it identified 12 significant gene profiles (gene clusters) comprised of 2,181 of the 4,503 adult sex-independent genes, and for 762 of the 1,212 adult sex-specific genes (Figure 3 and Additional file 4A-C). The DAVID annotation tool [37,38] was used to find significant enrichment clusters for each of the twelve STEM profiles; these results are presented in Additional file 5A-C. The largest adult sex-independent gene cluster, STEM profile 1 (Figure 3A), was comprised of 790 genes down regulated from 3 and 4 wk to 8 wk in both sexes and contained 39 significant DAVID enrichment clusters (Additional file 5A). The top three clusters showed very strong enrichment for genes of cell cycle, mitosis and cell division (41.8-fold enrichment), chromosome condensation (23.6-fold enrichment), and DNA damage and repair (11.6-fold enrichment), all consistent with the down regulation of cell division in adult compared to postnatal mouse liver [3]. The largest cluster of up regulated adult sex-independent genes (365 genes, STEM profile 2) was characterized by up regulation after 3 and 4 wk in both male and female liver (Figure 3B). Profile 2 contained two significant DAVID enrichment clusters, genes associated with responses to organic substances and endogenous stimuli, and ion and metal binding (Additional file 5A).

The largest cluster of adult sex-specific genes (276 male-specific genes; STEM profile 8), displayed strong up regulation from 3 and 4 wk to 8 wk in male liver, and weaker up regulation in female liver (Figure 3C). This profile contained three significant enrichment clusters, consisting of genes involved in pheromone binding and microsomal cytochrome P450/monooxygenase activity (Additional file 5B). The largest female-specific gene cluster (231 genes; STEM profile 11) was characterized by down regulation in male liver from 3 and 4 wk to 8 wk, without a change in female liver (Figure 3D). This profile contained 9 significant enrichment clusters, including cytochrome P450/monooxygenase activity, acyl CoA thioesterase, peroxisome, flavin monooxygenase, sulfotransferase, and lipid biosynthesis (Additional file 5C).

### HNF4α-dependence of developmentally responsive genes

To investigate whether the post-pubertal developmental changes that we observed are associated with a dependence of gene expression on HNF4α, a major regulator of liver-specific gene expression [25,40,41], we compared the gene groups showing up or down regulation during post-pubertal development to genes whose expression is either up or down regulated in response to liver-specific ablation of HNF4α [24]. In both male and female liver, genes up regulated from 3 wk to 8 wk showed highly significant enrichment for genes positively regulated by HNF4α (*p *= E-77 to E-110), and genes down regulated from 3 wk to 8 wk showed highly significant enrichment for the set of genes negatively regulated by HNF4α (*p *= E-66 to E-67) (Table 2). To determine whether adult sex-specific genes contribute to these enrichments, enrichment scores were calculated separately for male-specific and female-specific genes. Enrichment analysis was also calculated for stringent sex-independent genes, which were selected to exclude genes showing weak sex-specificity at 8 wk (see Materials and Methods). We found that male-specific genes contributed to the enrichment of the developmentally up regulated genes in genes positively regulated by HNF4α in both male liver (*p *= 8.8E-56) and female liver (*p *= 9.4E-09) (Additional file 6A). Male-specific genes also contributed to the enrichment of the genes down regulated developmentally in the set of genes negatively regulated by HNF4α in female liver (*p *= 5.6E-07), but not male liver (Additional file 6A). Female-specific genes contributed to the enrichment of the developmentally up regulated genes in genes positively regulated by HNF4α in female liver (*p *= 2.0E-27), and to the enrichment of developmentally down regulated genes in genes negatively regulated by HNF4α in male liver (*p *= 5.0E-38) (Additional file 6A). Stringent sex-independent genes showed the same patterns of enrichment that are seen for all genes in Table 2 (Additional file 6A). Moreover, significant overlap was seen between stringent sex-independent genes that are up regulated from 3 to 8 wk and genes that are positively regulated by HNF4α (66% overlap), and similarly, between stringent sex-independent genes that are down regulated from 3 to 8 wk and genes that are negatively regulated by HNF4α (65% overlap). Thus, both adult sex-independent and adult sex-specific genes are subject to positive regulation by HNF4α, in the case of the developmentally induced genes, and are subject to negative regulation by HNF4α, in the case of the developmentally repressed genes.

**Table 2:** Genes showing developmental changes are enriched for genes altered by liver-specific deletion of HNF4α

A. Background: All genes (22,831)	Genes up regulated in male HNF4α- deficient liver (2,619 genes)			Genes down regulated in male HNF4α- deficient liver (2,712 genes)		
	**# of overlapping genes**	**Fold enrichment**	**Fisher Test *p*-value**	**# of overlapping genes**	**Fold enrichment**	**Fisher Test *p*- value**

All genes up regulated in male liver 3-8 wk (1,550 genes)	*127*	*0.71*	*4.61E-05*	**537**	**2.92**	**3.87E-110**

All genes down regulated in male liver 3-8 wk (1,791 genes)	**486**	**2.37**	**1.15E-66**	*131*	*0.62*	*9.10E-10*

**B**. Background: All genes (22,831)	Genes up regulated in female HNF4α- deficient liver (2,227 genes)			Genes down regulated in female HNF4α- deficient liver (2,255 genes)		

	# of overlapping genes	Fold enrichment	Fisher Test *p*-value	# of overlapping genes	Fold enrichment	Fisher Test *p*- value

All genes up regulated in female liver 3-8 wk (1,087 genes)	93	0.88	0.208	**337**	**3.14**	**2.43E-77**

All genes down regulated in female liver 3-8 wk (1,360 genes)	**366**	**2.76**	**6.39E-67**	*85*	*0.63*	*4.35E-06*

Genes identified in the present microarray study were grouped based on their regulation from 3 wk to 8 wk and compared to genes from a previous microarray study [24] grouped by their response to liver-specific HNF4α-deficiency. All of the genes used to calculate the enrichment scores shown in this Table were represented on both microarray platforms. Total numbers of genes comprising each group are shown in parenthesis. Sample enrichment score calculation: [% of the genes down regulated in male liver from 3 wk to 8 wk that are up-regulated in male HNF4α-knockout mouse liver]/[% of all genes that are up-regulated in male HNF4α-knockout mouse liver], i.e., (486/1,791)/(2,619/22,831) = 2.37 fold enrichment. **Bold **text indicates enrichment with a highly significant *p*-value (*p *< 0.0001, Fisher's exact test), and *italic *text indicates depletion with a highly significant *p*-value (*p *< 0.0001, Fisher's exact test).

### Pituitary dependence of developmentally responsive genes

Growth hormone (GH) is an established regulator of a large majority of sex-specific gene expression in adult mouse and rat liver [5]. At 3 wk of age, serum concentrations of GH are low and subsequently increase in both male and female mice [42,43], with the temporal pattern of pituitary GH secretion becoming sexually dimorphic by 8 wk of age [44,45]. To investigate whether the observed developmental changes in liver gene expression might reflect changes in secretion of GH or other pituitary-dependent hormones, we compared the sets of genes that are up or down regulated from 3 to 8 wk of age to the sets of genes whose expression is altered following hypophysectomy [27]. Genes that were down regulated in either sex from 3 to 8 wk showed the strongest enrichment for genes up regulated following hypophysectomy, i.e., genes negatively regulated by pituitary hormone (*p *= E-24 to E-47) (Table 3). Conversely, genes up regulated from 3 to 8 wk showed the strongest enrichment for genes positively regulated by pituitary hormone (*p *= E-16 to E-30) (Table 3). This suggests that developmental changes in pituitary hormones, or pituitary-dependent hormones, play a significant role in the observed developmental changes in gene expression. Enrichment scores calculated separately for male-specific, female-specific, and stringent sex-independent genes showed that male-specific genes contributed to the enrichment of the developmentally up regulated genes in genes positively regulated by pituitary hormone in male liver, whereas female-specific genes contribute to the enrichment seen for the down regulated genes in male liver and to the enrichment seen for the up regulated genes in female liver (Additional file 6B). In contrast, the stringent sex-independent genes that were developmentally up regulated showed enrichment for genes under negative pituitary regulation (*p *= E-10 to E-18), suggesting that their up regulation from 3 to 8 wk reflects the loss of an inhibitory pituitary factor (Additional file 6B).

**Table 3:** Genes showing developmental changes are enriched for genes altered by hypophysectomy

A. Background: All genes (22,635)	Genes up regulated in male hypophysectomized liver (2,250 genes)			Genes down regulated in male hypophysectomized liver (2,202 genes)		
	**# of overlapping genes**	**Fold enrichment**	**Fisher Test *p*-value**	**# of overlapping genes**	**Fold enrichment**	**Fisher Test *p*-value**

All genes up regulated in male liver 3-8 wk (1,542 genes)	**213**	**1.39**	**3.05E-06**	**306**	**2.04**	**2.13E-30**

All genes down regulated in male liver 3-8 wk (1,773 genes)	**393**	**2.23**	**7.12E-47**	163	0.95	0.479

**B**. Background: All genes (22,635)	Genes up regulated in female hypophysectomized liver (1,521 genes)			Genes down regulated in female hypophysectomized liver (1,535 genes)		

	# of overlapping genes	Fold enrichment	Fisher Test *p*-value	# of overlapping genes	Fold enrichment	Fisher Test *p*-value

All genes up regulated in female liver 3-8 wk (1,070 genes)	**131**	**1.82**	**2.38E-10**	**151**	**2.08**	**2.91E-16**

All genes down regulated in female liver 3-8 wk (1,364 genes)	**203**	**2.21**	**5.87E-24**	**139**	**1.50**	**5.16E-06**

Genes identified in the present microarray study were grouped based on their regulation from 3 wk to 8 wk and compared to genes from a previous microarray study [27] grouped by their response to hypophysectomy. Enrichments were calculated as described in Table 2. Total numbers of genes comprising each group are shown in parenthesis. **Bold **text indicates enrichment with a highly significant *p*-value (*p *< 0.0001, Fisher's exact test).

### Developmental changes in expression of transcriptional regulators

We sought to identify transcription factors that could potentially contribute to the age dependent changes in gene expression described above. 323 of the 4,503 adult sex-independent genes were identified as potential transcriptional regulators by their Gene Ontology (GO) descriptors. 37 of these genes showed at least a 2-fold change in expression on all arrays that had a significant *p*-value (*p *< 0.0001) (Additional file 7A). DAVID analysis of the 37 genes identified 12 significant clusters, 5 of which were closely associated with transcription. The other 7 clusters consisted of genes that contain a basic motif, or are involved in circadian rhythm, chromosomal organization, DNA replication, DNA repair or metal ion binding (Additional file 7B).

Using a similar approach, 23 male-specific genes and 32 female-specific genes were identified as potential transcriptional regulators. Within these groups, 3 male-specific and 6 female-specific genes had a sex-bias of at least 2-fold at 8 wk and were confirmed as showing sex-specific expression in earlier mouse liver microarray studies (Table 4) [27,32]. Four of these transcriptional regulators did not show sex-specific expression until 8 wk (*Id1, Jazf1, Klf17, Ybx2*) while the other five displayed sex-specificity by 4 wk (*Cdx4, Cux2, Ihh, Tox, Trim24*). The three male-specific transcriptional regulators (*Ihh, Klf17, Ybx2*) were up regulated from 3 wk to 8 wk in male liver, while *Ybx2 *was also up regulated from 3 to 8 wk in female liver. All 6 female-specific transcriptional regulator genes were down regulated in male liver after 3 or 4 wk, but in female liver showed no change in expression (*Cdx4, Jazf1, Id1*) or were up regulated from 3 or 4 wk to 8 wk (*Cux2, Tox, Trim24*).

**Table 4:** Sex-specific transcriptional regulators

Gene Name	*Ihh*	*Klf17*	*Ybx2*	*Cdx4*	*Cux2*	*Id1*	*Jazf1*	*Tox*	*Trim24*
Accession Number	NM_010544	NM_029416	NM_016875	NM_007674	NM_007804	NM_010495	NM_173406	NM_145711	NM_145076

Sex specificity	M	M	M	F	F	F	F	F	F

Onset of sex specificity	4 wk	8 wk	8 wk	4 wk	4 wk	8 wk	8 wk	4 wk	4 wk

Developmental change in M liver (3, 4 wk to 8 wk)	up	up	up	down	down*	down	down	down*	down

Developmental change in F liver (3, 4 wk to 8 wk)	no change	no change	up	no change	up	no change	no change	Up	up*

Response to HNF4α-knockout	down in M	down in M	-	up in M	up in M	up in M up in F	up in M up in F	up in M up in F	up in M up in F

Response to STAT5-knockout	-	-	down in M	no effect	up in M	-	no effect	up in M	up in M

Response to Hypox	down in M	down in M	-	down in F	up in M, down in F	up in M	up in M	up in M	down in F

Response to continuous GH	-	-	down in M at ≥ 4 day	up in M at ≥ 4 day	up in M at ≥ 2 day	-	no effect	no effect	up in M at ≥ 10 hr

Adult sex-specific genes with a |fold change| > 2 and *p *< 0.0001 for the male 8 wk vs. female 8 wk microarray comparison and having GO descriptors that contain the words "DNA binding" or "transcription" were identified as potential transcriptional regulators. The transcription factor genes listed in this table were previously found to be altered in expression in response to hypophysectomy [27], STAT5b-deficiency [32], HNF4α-deficiency [24], or treatment with continuous GH [46]. M, male, F, female

* Indicates a gene that displayed a developmental change at 4 wk but not 3 wk

- Indicates no probe for the gene on the microarray platform that was used for the analysis.

### qPCR validation

**Figure 4:**
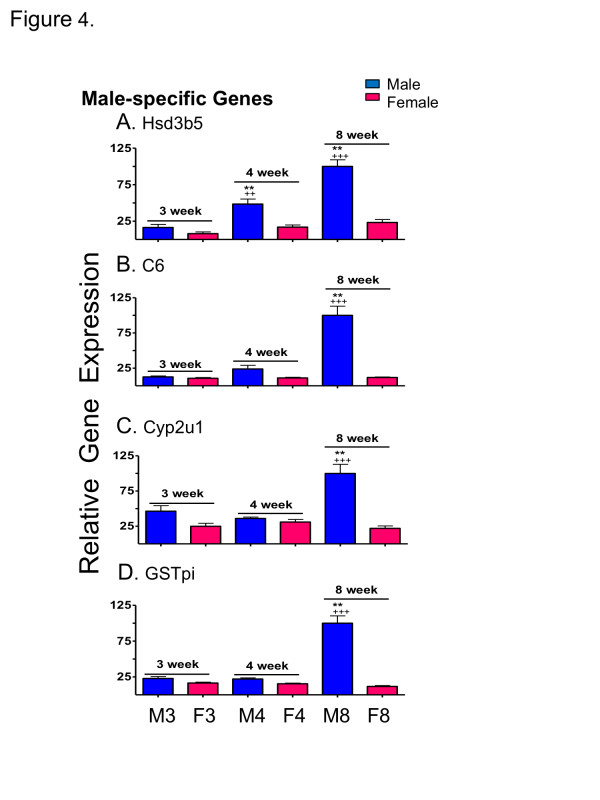
**qPCR analysis of male-specific genes**. qPCR analysis of relative RNA levels in male (M) and female (F) livers at 3, 4 and 8 wk, as marked at the bottom. Data shown for the four indicated adult male-specific genes are normalized to the 18S RNA of each sample, and based on n = 9-10 individuals per group (mean ± SEM). Statistical analysis (two-way ANOVA with Bonferroni post tests) was as follows: * *p *< 0.01, and ** *p *< 0.001, for male vs. female comparisons at each age; + *p *< 0.05, ++ *p *< 0.01, and +++ *p *< 0.001, for 4 wk vs. 3 wk, and for 8 wk vs. 4 wk comparisons within each sex. Very similar results were obtained by microarray analysis (Additional file 8).

**Figure 5:**
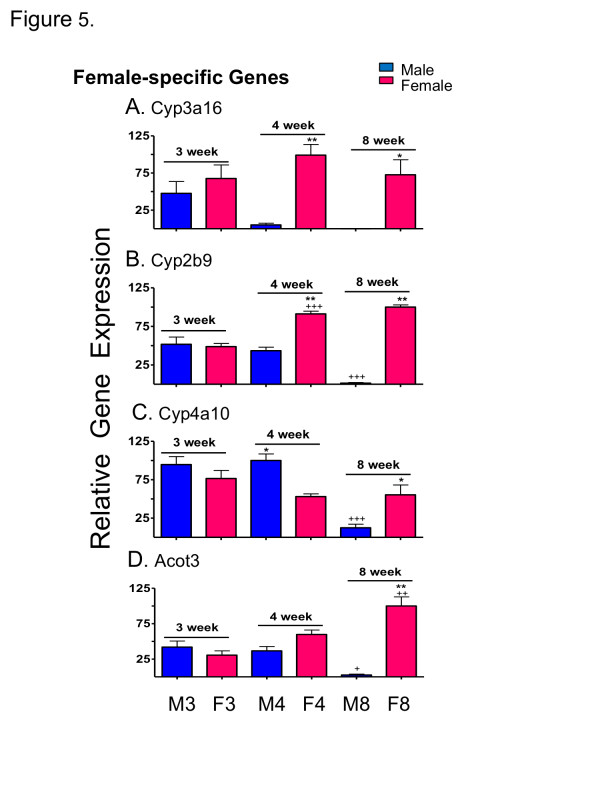
**qPCR analysis of female-specific genes**. qPCR analysis of relative RNA levels in male (M) and female (F) livers at 3, 4 and 8 wk. Data shown for the four indicated adult female-specific genes are normalized to the 18S RNA of each sample, and based on n = 9-10 individuals (mean ± SEM). Statistical analysis was as described in Figure 4. Very similar results were obtained by microarray analysis (Additional file 9).

qPCR was used to validate the results obtained from the above-described developmental patterns for a selected group of sex-specific genes. One of the four adult male-specific genes investigated (*Hsd3b5*) was expressed at a significantly higher level in male than female liver by 4 wk and significantly increased with age in male liver (*p *< 0.01 from 3 wk to 4 wk and *p *< 0.001 from 4 wk to 8 wk) (Figure 4A). The other three genes (*C6, Cyp2u1, Gstp*) did not show significant sex differences in expression until 8 wk (*p *< 0.001) (Figure 4B-D). Two of the adult female-specific genes examined (*Cyp3a16, Cyp2b9*) displayed sex-specificity at 4 wk (*p *< 0.001) (Figure 5A, B), while a third gene (*Cyp4a10*) showed significant male-bias at 4 wk (*p *< 0.01) (Figure 5C). All four genes displayed significant female-biased expression at 8 wk, as expected (Figure 5A-D). Two-way ANOVA analysis indicated significant (*p *< 0.0001) interaction between age and sex for all genes examined, except *Cyp3a16*. These qPCR profiles were remarkably similar to the corresponding microarray profiles (Additional file 8, Additional file 9) except in the case of *Cyp3a16*, where the microarray probe indicated a much lower sex-difference due to its substantial cross-hybridization with *Cyp3a11*, an adult sex-independent gene.

## Discussion

The present study of genome-wide transcriptional profiles in mouse liver was conducted to identify developmental changes that occur from 3 wk (weaning) to 4 wk (just prior to puberty) to 8 wk of age (young adulthood). During this period of development the liver is completing its final stages of growth and liver function is changing from hematopoiesis to regulation of metabolism and other biological processes, including bile secretion, xenobiotic metabolism, and cholesterol homeostasis [3,8,47]. Genes involved in growth, cell cycle, and DNA replication were found to be down regulated after 3 wk and 4 wk, while genes associated with specialized liver functions such as drug metabolism and inflammatory response were up regulated. The latter findings are similar to another study where down regulation of genes associated with mitosis, DNA replication, RNA splicing, and transcription was seen at postnatal days 7, 14, 21 and 126 compared to the mean expression level determined at 14 different time points, beginning in embryonic development [3]. Additionally, extensive changes in the expression of adult sex-specific genes were observed, especially in male liver, where the majority of male-specific genes were up regulated and nearly half of female-specific genes were down regulated. Developmental changes in adult sex-independent genes were also more extensive in male liver compared to female liver.

Genes up regulated from 3 to 8 wk were significantly enriched in the set of genes positively regulated by the liver transcription factor HNF4α, as determined by their response to liver-specific deletion of HNF4α [24], while genes down regulated during this developmental period showed significant enrichment for genes negatively regulated by HNF4α. However, some differences in HNF4α regulation of the developmentally regulated genes were seen between male and female liver (Table 5). The positive effects of HNF4α on developmentally up regulated female-specific genes were only apparent in female liver, while negative effects of HNF4α on developmentally down regulated genes were associated with male-specific genes in female liver and with female-specific genes in male liver. Consistent with these findings, binding sites for HNF4α are overrepresented in genes that are up regulated at postnatal day 7, 14, 21, and 126 compared to the mean expression value at 14 developmental time points [3]. The highly significant association shown here between developmental up regulation and positive regulation by HNF4α, and between developmental down regulation and negative regulation by HNF4α suggests that the expression or activity of HNF4α or an HNF4α-dependent factor(s) increases in mouse liver from 3 wk to 8 wk. However, no change in HNF4α mRNA levels between 3 wk and 8 wk was seen on our microarrays. HNF4α protein and mRNA are both induced at birth and increase to adult-like levels within 2 weeks, at which time expression of HNF4α7, an alternative isoform with a unique N-terminal protein sequence is extinguished [48]. Further study is required to elucidate the mechanisms by which HNF4α contributes to the developmental changes of both sex-specific and sex-independent genes.

**Table 5:** Summary of the proposed role of HNF4α and pituitary hormone in developmental changes in liver gene expression

	Developmental change	Male liver (3 wk to 8 wk)	Female liver (3 wk to 8 wk)
Adult male-specific genes	Up	HNF4α (+) Pituitary hormone (+)	HNF4α (+)
	
	Down	No regulation	HNF4α (-)

Adult female-specific genes	Up	No regulation	HNF4α (+) Pituitary hormone (+)
	
	Down	HNF4α (-) Pituitary hormone (-)	No regulation

Adult sex-independent genes	Up	HNF4α (+) Pituitary hormone (-)	HNF4α (+) Pituitary hormone (+, -)
	
	Down	HNF4α (-)	HNF4α (-)

This table summarizes the findings presented in Table 2, Table 3, and Additional files 6A-B. A plus sign indicates positive regulation and a minus sign indicates negative regulation. In male liver, HNF4α and pituitary hormone positively regulate male-specific genes that are up regulated from 3 to 8 wk; both factors also negatively regulate female-specific genes that are down regulated from 3 to 8 wk. In female liver, HNF4α positively regulates developmentally up regulated male-specific and female-specific genes, while negatively regulating developmentally down regulated male-specific genes. HNF4α and pituitary hormone exhibited similar patterns of regulation of sex-independent genes in male as in female liver, except that pituitary hormone showed both positive regulation and negative regulation of the developmentally up regulated sex-independent genes in female liver only

Comparison of the genes undergoing developmental changes to the set of genes whose expression changes in mouse liver following hypophysectomy [27] revealed differences in the regulation by pituitary hormone between male and female liver (Table 5). In male liver, pituitary hormone positively regulates male-specific genes that are up regulated from 3 wk to 8 wk, while negatively regulating female-specific genes that are down-regulated during the same time period. In contrast, in female liver, pituitary hormone positively regulated female-specific genes up regulated during female development but did not show significant enrichment for effects on male-specific genes. These differences in pituitary hormone regulation of sex-specific genes in male vs. female liver could be explained by the sex differences in pituitary GH secretion patterns, which are known to regulate many sex-dependent genes in the liver [5]. We also observed pituitary hormone regulation of the developmentally regulated stringent sex-independent genes, with negative regulation by pituitary hormone apparent in male liver, and both positive and negative regulation apparent in female liver (Table 5). The latter finding could be explained by increased secretion of a negative regulatory factor after 3 wk of age, or by decreased secretion of a positive regulatory factor. One such candidate factor is corticosterone, whose adrenal production is stimulated by adrenocorticotropic hormone (ACTH) produced by the anterior pituitary gland, and has ~3 times higher plasma concentrations in 20 day old mice compared to adult male mice [49].

We sought to identify transcriptional regulators that undergo developmental changes in mouse liver, as these could serve as regulators of the developmental changes in RNA transcripts described here. Seven developmentally regulated adult sex-independent transcriptional regulators (*Asf1b, Hells, Hmgb2, Padi4, Ppard, Prim2, Top2a*) are associated with chromosomal organization and were down regulated from 3-4 wk to 8 wk. One or more of these transcription factors could be associated with the down regulation of cell cycle and mitosis that occurs in liver from the postnatal period to puberty [8]. Seven other adult sex-independent transcriptional regulators identified here (*Arntl, Cry1, Dbp, Nr1d1, Per2, Per3, Tef*) help establish circadian rhythms. Many genes are expressed in a circadian manner in the liver, most notably genes active in drug metabolism and bile acid synthesis, including sex-specific genes [50-52]. A related gene, *Per1*, changes in expression at postnatal day 22 in rat liver [53]. Another study found that clock-associated genes become rhythmic by postnatal day 30 [54].

Prior studies of sex-specific hepatic gene expression have primarily focused on the adult period. Presently, excluding Y-chromosome genes, we found only 13 adult sex-specific genes that displayed sex-specificity at 3 wk and retained their sex specificity at 4 wk and 8 wk of age. By 4 wk, an additional 104 genes showed female-specific expression and an additional 54 genes displayed male-specific expression. Five of the 158 genes that displayed their adult sex-specificity at 4 wk of age encode transcriptional regulators (*Cdx4, Cux2, Ihh, Tox, Trim24*); these genes could contribute to the developmental changes leading to global acquisition of liver sex-specificity by 8 wk. Consistent with our finding in mouse liver, a microarray study of gene expression in postnatal rat liver (ages ranging from 2 wk to 104 wk) reported very few sex-specific genes at 2 wk and 5 wk. Moreover, there was a large increase in the number of sex-specific genes, including many genes associated with drug metabolism, by 8 wk [55].

The major increase in sex-specific gene expression between 4 wk and 8 wk of age shown here for mouse liver can in part be explained by the developmental changes in growth hormone (GH) secretion during this developmental period. GH has an established role in regulating sex-specific gene expression in mouse liver [4-6], and the sex-specific patterns of pituitary GH secretion are imprinted during the neonatal period but are not manifested until puberty [6,42,56]. *CYP3A4 *shows female-biased expression in human liver [57] and displays a similar postnatal development expression pattern in mouse liver when it is introduced as a transgene [20,58]. This suggests that the genomic sequences that dictate the observed pattern of developmental repression in male liver are conserved between mouse and human. In the present study, the change from sex-independent expression at 3-4 wk to sex-specific expression at 8 wk was closely associated with the up regulation of male-specific genes and the down regulation of female-specific genes in male liver. Conversely, in female liver the most frequent change was one that occurred in both male and female liver. Since GH is known to be the major hormonal regulator of these sex-specific genes, the developmental patterns that we observed suggest that the male-specific GH pattern could either be turning on a transcriptional activator or turning off a transcriptional repressor to up regulate male-specific gene expression. The male-specific GH pattern could also down regulate female-specific genes in male liver by either turning on a transcriptional repressor or by turning off a transcriptional activator. STAT5b and HNF4α are essential transcriptional regulators of sex-specific liver gene expression, and sex-specific genes are enriched for genes that are affected by deletion of STAT5b or HNF4α [24,32,39,46]. However, activation of STAT5 alone is not sufficient to induce male-specific gene expression in pre-pubertal rats [59], indicating that other developmentally regulated factors, such as the 9 sex-specific transcription factors identified in this study, may be required to achieve sex-specific gene expression. Five of the 9 factors displayed developmental changes in male liver only (Table 4), and could contribute to the selective up regulation of male-specific genes and/or down regulation of female-specific genes seen in male liver but not female liver. The three male-specific transcription factor genes of interest are transcriptional activators. Y-box protein 2 (*Ybx2*) is an RNA-binding protein in germ cells but also has the ability to bind to and stimulate transcription of the mouse protamine-2 promoter [60]. Indian hedgehog (*Ihh*) plays a role in endodermal differentiation and can activate gene transcription by binding to Patched receptors Ptc1 and Ptc2 [61]. Finally, Kruppel-like factor 17 (*Klf17*) is a member of the small protein zinc finger family and can activate transcription from CACCC-box elements [62].

The six female-specific transcriptional regulators identified here are either known transcriptional repressors or their function is unknown. Cut-like homeobox 2 (*Cux2*) is a member of the cut/homeodomain family of transcription factors and can act as a transcriptional repressor [63]. Caudal type homeobox 4 (*Cdx4*) is a homeodomain transcription factor that may play a role in hematopoiesis [64]. Thymus high-mobility group box protein (*Tox*) is a member of the sequence independent high mobility group (HMG) box family and a regulator of differentiation of developing T-cells [65]. Tripartite motif-containing 24 (*Trim24*) contains a zinc binding motif, a coiled-coiled region, and a RING domain, which has been shown to act as an E3-ubiquitin ligase and target tumor suppressor p53 for degradation [66]. Juxtaposed with another zinc finger protein 1 (*Jazf1*) contains three zinc finger motifs and is of unknown function but is associated with lipid metabolism, diabetes mellitus, and prostate cancer [67]. Finally, inhibitor of DNA binding 1 (*Id1*) functions as a negative regulator of basic helix-loop-helix (bHLH) transcription factors and is trans-activated by JAK/STAT5 signaling in erythroid cells [68].

Three of the female-specific genes (*Cux2, Tox, Trim24*) were previously characterized as potential regulators of sex-specific gene expression [21]. *Cux2 *expression is highly female-specific in both mouse and rat liver [21]. Binding sites for Cux1/Cux2 are statistically overrepresented at or near STAT5b-dependent male-specific genes, suggesting that Cux2 could be acting as a repressor of male-specific gene expression in female liver [21]. Further characterization of Cux2 and the other sex-specific transcriptional regulators is required to ascertain their contributions to sex-specific liver gene expression.

Overall, the observed changes in liver gene expression from the pre-pubertal period to young adulthood reflect the deceleration of liver growth and the induction of specialized liver functions. The number of sex-biased genes expressed during this period also increased dramatically at this time. Widespread changes in both sex-independent and sex-biased genes were observed, and primarily occurred in male liver. This male bias in these gene expression changes may be due to differences in pituitary hormone secretion and/or regulation by HNF4α.

## Competing interests

The authors declare that they have no competing interests.

## Authors' contributions

TLC carried out all of the laboratory studies and data analysis and contributed to writing all sections of the paper. DJW acted as mentor for study design, data analysis, and contributed to writing and editing all sections of the paper. All authors read and approved the final version of the manuscript.

## Supplementary Material

Additional file 1 **qPCR primers**. Primers used for qPCR validation of microarray results for selected male-specific (*Cyp2u1, C6, GSTpi, Hsd3b5*) and female specific (*Acot3, Cyp2b9, Cyp3a16, Cyp4a10*) genes. Click here for file

Additional file 2 **Explanation of total flagging sum (TFS) classification of regulated microarray gene groups**. A 7 decimal point TFS number is assigned to each gene (microarray probe) represented on the microarray based on the pattern of regulation that the gene exhibits across the set of 7 microarrays. Each of the 7 digits to the right of the decimal point place represents one the 7 microarrays, numbered sequentially from left to right, as indicated. A value of 0 indicates the gene does not meet the conditions for significant differential gene expression (as defined in Methods) for that microarray, a value of 1 indicates up regulation, and a value of 2 indicates down regulation. In the example shown, for a gene assigned TFS number 28.0022200, the 1^st ^and 2^nd ^decimal places are both 0, indicating that the gene is sex-independent at 3 and 4 wk. The 3^rd ^decimal places is 2, indicating that the gene is female-specific at 8 wk. The 4^th ^and 5^th ^decimal places are 2, indicating that the gene is down regulated from 3 and 4 wk to 8 wk in male liver. The 6^th ^and 7^th ^decimal places are 0, indicating that there is no regulation of the gene from 3 and 4 wk to 8 wk in female liver. Each decimal place is also assigned a binary flag value: 1^st ^decimal place = 1, 2^nd ^decimal place = 2, 3^rd ^decimal = 4, 4^th ^decimal = 8, 5^th ^decimal = 16, 6^th ^decimal = 32, and 7^th ^decimal = 64. The whole number portion of the TFS is calculated by adding the binary flag value of each decimal place representing a microarray that met the thresholds for significance. Thus, the whole number portion of the TFS number is calculated as 28 = 4 (3^rd ^decimal place) + 8 (4^th ^decimal place) + 16 (5^th ^decimal place). Click here for file

Additional file 3 **A.** Number of differentially expressed (regulated) genes in each of the seven microarray comparisons. Each gene listed in this table meets the threshold requirements of a |fold change| > 1.5, *p *< 0.0001, and has a well above background intensity value for at least one of the seven microarray comparisons. The number of sex-specific genes greatly increased from 3 wk to 8 wk; also see Table 1. A larger number of genes changed from 3 or 4 wk to 8 wk in male liver compared to female liver. **B**. Detailed microarray data for each of the 5,715 regulated genes. Each gene listed in this table meets the threshold requirements as in Additional file 3A. Data shown include Rosetta-computed weighted ratios, Rosetta *p*-values, and microarray signal intensities for the 7 microarray experiments. The "is well above background" value column uses a binary value to identify arrays that met this requirement (score = 1), as well as arrays where this requirement was not met (score = 0.1 or 0.01), in which case the probe is considered as not meeting the threshold for significance for that array, irrespective of the fold-change and p-values shown. Stringent sex-independent genes meet the criteria of a |fold change| < 1.2, a *p*-value > 0.01, and a minimum intensity of 25. Genes that do not meet this criteria and do not meet the criteria of sex-specificity (|fold change| > 1.5 and *p*-value < 0.0001) are labeled as sex-independent. **C**. Developmental changes in expression during pre-pubertal period. The gene changes listed take into account the developmental changes occurring from both 3 wk to 8 wk and from 4 wk to 8 wk and are based on the TFS number assigned to each gene (Additional file 2). The most prominent developmental change observed was a change in male liver only. A larger percentage of genes displayed a developmental change in male liver (77%) than in female liver (53%). Click here for file

Additional file 4 **A. Box and whisker plots representing gene expression patterns in STEM cluster profiles 9, 10, and 12**. Boxes represent the 25^th ^to the 75^th ^percentile of gene expression ratios for each of the 7 microarray comparisons indicated below the X-axis. A horizontal line across each box indicates the median expression ratio. The whiskers that extend above and below each box represent the highest and the lowest values. M3, male at 3 wk; M4, male at 4 wk; M8, male at 8 wk; F3, female at 3 wk; F4, female at 4 wk; and F8, female at 8 wk. (A) Profile 9 is comprised of male-specific genes that are up regulated from 3 wk to 8 wk and from 4 wk to 8 wk in male liver but not in female liver. (B) Profile 10 is comprised of male-specific genes that are, on average, slightly up regulated from 3 wk to 8 wk and from 4 wk to 8 wk in male liver and slightly down regulated from 3 wk to 8 wk in female liver. (C) Profile 12 is comprised of female-specific genes that are up regulated from 3 wk to 8 wk and from 4 wk to 8 wk in female liver. **B**. Box and whisker plots representing gene expression patterns in STEM cluster profiles 3, 4, and 5. Graphs are presented as in Additional file 4A. (A) Profile 3 is comprised of sex-independent (SI) genes that are up regulated from 3 wk to 8 wk and from 4 wk to 8 wk in both male and female liver. (B) Profile 4 is comprised of sex-independent genes that are up regulated from 3 wk to 8 wk and from 4 wk to 8 wk in male liver. (C) Profile 5 is comprised of sex-independent genes that are up regulated from 3 wk to 8 wk and from 4 wk to 8 wk in female liver, but show weak up regulation in male liver. **C**. Box and whisker plots representing gene expression patterns in STEM cluster profiles 6 and 7. Graphs are presented as in Additional file 4A. (A) Profile 6 is comprised of sex-independent (SI) genes that are down regulated from 3 wk to 8 wk in both male and female liver and from 4 wk to 8 wk in male liver. (B) Profile 7 is comprised of sex-independent genes that are down regulated from 3 wk to 8 wk and 4 wk to 8 wk in male liver, while slightly up or down regulated from 3 wk to 8 wk in female liver. Click here for file

Additional file 5 **Significant DAVID functional annotation clusters found for each STEM cluster**. Each STEM cluster was analyzed using the DAVID annotation tool. DAVID annotation clusters with a minimum enrichment score of 1.3 (equivalent to a *p*-value of 0.05) for the genes in each STEM profile are shown, listed in descending order of enrichment score. The *p*-values (also referred to as the Ease Score) are derived from a one-tail Fisher Exact Test. **A**. DAVID functional annotation clusters for the 7 sex-independent STEM clusters. **B**. DAVID functional annotation clusters for the 3 male-specific STEM clusters. **C**. DAVID functional annotation clusters for the 2 female-specific STEM clusters. Click here for file

Additional file 6 **A.** Genes showing developmental changes are enriched for genes altered by liver-specific deletion of HNF4α. Shown here is the enrichment information presented in Table 2 for all genes, as well as the additional enrichment scores calculated separately for male-specific, female-specific, and stringent sex-independent genes. Stringent sex-independent genes had a |fold-change| < 1.2, a *p*-value > 0.01, and a microarray intensity ≥ 25. All genes were used as the background gene set when calculating the enrichment scores for male-specific, female-specific, and all genes. All stringent sex-independent genes were used as the background when calculating the enrichment scores for stringent sex-independent genes. Fold enrichments (i.e., enrichment scores) were calculated as described in Table 2. The results presented in this table show which groups of genes are contributing to the significant enrichment scores reported based on an analysis of all genes in Table 2. For example, the enrichment between all genes down-regulated in male liver from 3 wk to 8 wk and genes up-regulated in HNF4α KO male liver (2.37-fold, *p*-value 1.15E-66) (Table 2) is seen in this table to be due to contributions from female-specific genes (3.7-fold, *p*-value 4.98E-38) and stringent sex-independent genes (1.92-fold, *p*-value 6.23E-15), but not male-specific genes (1.65-fold, *p*-value 0.418). **B**. Genes showing developmental changes are enriched for genes altered by hypophysectomy. This table shows the enrichment data presented in Table 3 for all genes, together with enrichment scores calculated separately using male-specific, female-specific, and stringent sex-independent genes. Stringent sex-independent genes had a |fold-change| < 1.2, a *p*-value > 0.01, and a microarray signal intensity ≥ 25. The set of all genes common to both microarray platforms was used as the background when calculating the enrichment scores for male-specific, female-specific, and all genes. The set of all stringent sex-independent genes was used as the background when calculating the enrichment scores for stringent sex-independent genes. Enrichments scores were calculated as described in Table 3. The results presented in this table show which groups of genes (male-specific, female-specific and stringent sex-independent genes) are contributing to the significant enrichment scores reported based on an analysis of all genes in Table 3. For example, the enrichment between all genes down regulated in male liver from 3 wk to 8 wk and genes up regulated in hypophysectomized male liver (2.23-fold, *p*-value 7.12E-47) (Table 3) is seen in this table to be due to contributions from female-specific genes (enrichment score = 4.03-fold, *p*-value 4.40E-37) but not from male-specific genes (enrichment = 0.67-fold, *p*-value 1) or from stringent sex-independent genes (enrichment = 1.51-fold, *p*-value .0001277), neither of which show significant enrichment. Click here for file

Additional file 7 **A.** Sex-independent transcriptional regulators. Listed are 37 sex-independent genes with a |fold change| > 2-fold for each array that had a *p*-value < 0.0001 and GO descriptors that contained the words "DNA binding" and/or "transcription". These genes are considered to be candidate transcriptional regulators of the developmentally regulated genes. **B**. Significant DAVID functional annotation clusters identified for the 37 sex-independent transcriptional regulators. Shown here are DAVID annotation clusters with a minimum enrichment score of 1.3 (equivalent to a *p*-value of 0.05), listed in descending order of cluster enrichment score. The *p*-values (also referred to as the Ease Score) are derived from a one-tail Fisher Exact Test. Click here for file

Additional file 8 **Microarray intensities of male-specific genes**. These graphs present the relative microarray intensities of each indicated male-specific gene. Expression patterns are very similar to those determined by qPCR analysis in Figure 4. Click here for file

Additional file 9 **Microarray intensities of female-specific genes**. These graphs present the relative microarray intensities of each indicated female-specific gene. Expression patterns are very similar to those determined by qPCR analysis in Figure 5. The exception is *Cyp3a16*, whose microarray probe cross-hybridizes with *Cyp3a11*, a non-sex-specific gene, which results in a much lower sex-difference that that observed by qPCR. Click here for file
