# Supplementary material for: Sex-specific mouse liver gene expression: genome-wide analysis of developmental changes from pre-pubertal period to young adulthood
Source: Biol Sex Differ. 2012 Apr 4;3:9. doi: 10.1186/2042-6410-3-9 (PMC3350426; doi:10.1186/2042-6410-3-9)
Supplement: Additional file 2 — Explanation of total flagging sum (TFS) classification of regulated microarray gene groups. A 7 decimal point TFS number is assigned to each gene (microarray probe) represented on the microarray based on the pattern of regulation that the gene exhibits across the set of 7 microarrays. Each of the 7 digits to the right of the decimal point place represents one the 7 microarrays, numbered sequentially from left to right, as indicated. A value of 0 indicates the gene does not meet the conditions for significant differential gene expression (as defined in Methods) for that microarray, a value of 1 indicates up regulation, and a value of 2 indicates down regulation. In the example shown, for a gene assigned TFS number 28.0022200, the 1st and 2nd decimal places are both 0, indicating that the gene is sex-independent at 3 and 4 wk. The 3rd decimal places is 2, indicating that the gene is female-specific at 8 wk. The 4th and 5th decimal places are 2, indicating that the gene is down regulated from 3 and 4 wk to 8 wk in male liver. The 6th and 7th decimal places are 0, indicating that there is no regulation of the gene from 3 and 4 wk to 8 wk in female liver. Each decimal place is also assigned a binary flag value: 1st decimal place = 1, 2nd decimal place = 2, 3rd decimal = 4, 4th decimal = 8, 5th decimal = 16, 6th decimal = 32, and 7th decimal = 64. The whole number portion of the TFS is calculated by adding the binary flag value of each decimal place representing a microarray that met the thresholds for significance. Thus, the whole number portion of the TFS number is calculated as 28 = 4 (3rd decimal place) + 8 (4th decimal place) + 16 (5th decimal place). [file 2042-6410-3-9-S2.PDF]

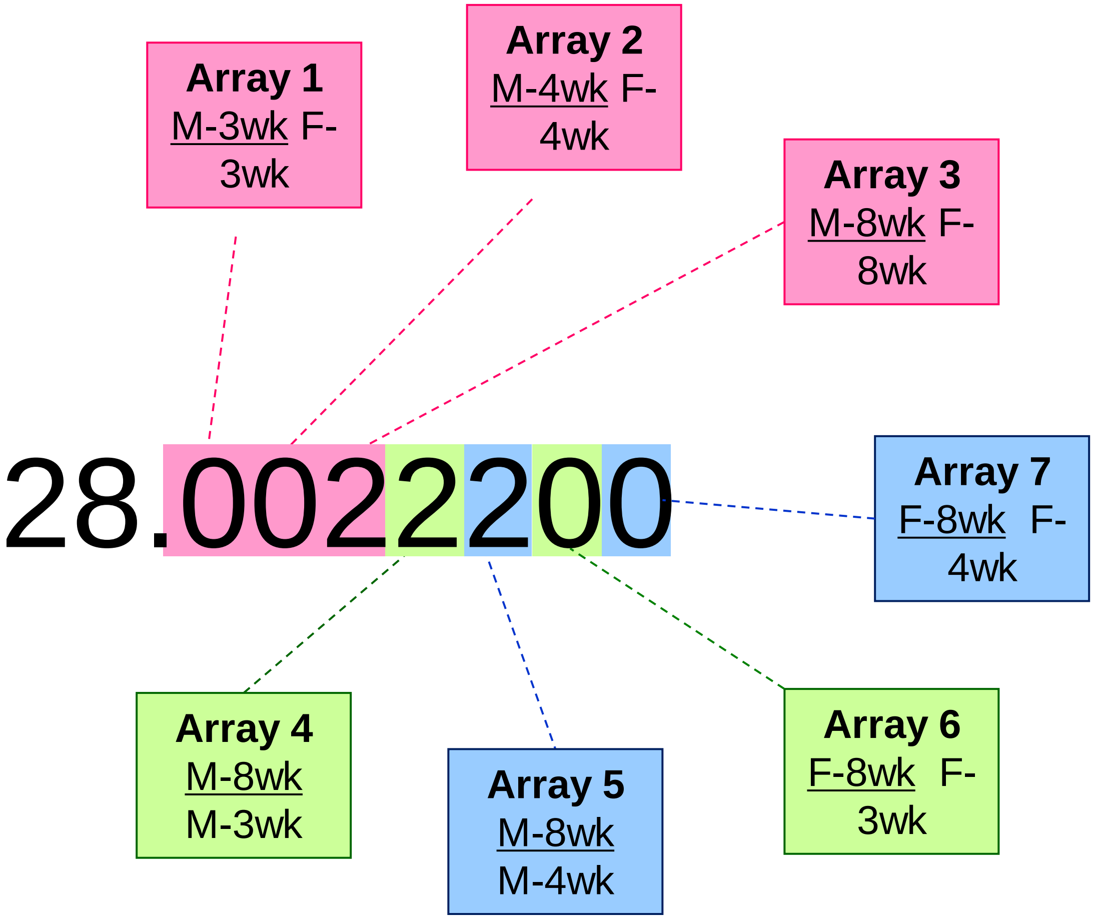

TFS decimal digit = 1 for male-specificity or up-regulation
TFS decimal digit = 2 for female-specificity or down-regulation

Sex specificity

Change in Expression 3wk to 8wk

Change in Expression 4wk to 8wk
