# Supplementary material for: Sex-specific mouse liver gene expression: genome-wide analysis of developmental changes from pre-pubertal period to young adulthood
Source: Biol Sex Differ. 2012 Apr 4;3:9. doi: 10.1186/2042-6410-3-9 (PMC3350426; doi:10.1186/2042-6410-3-9)

Additional File 4A.

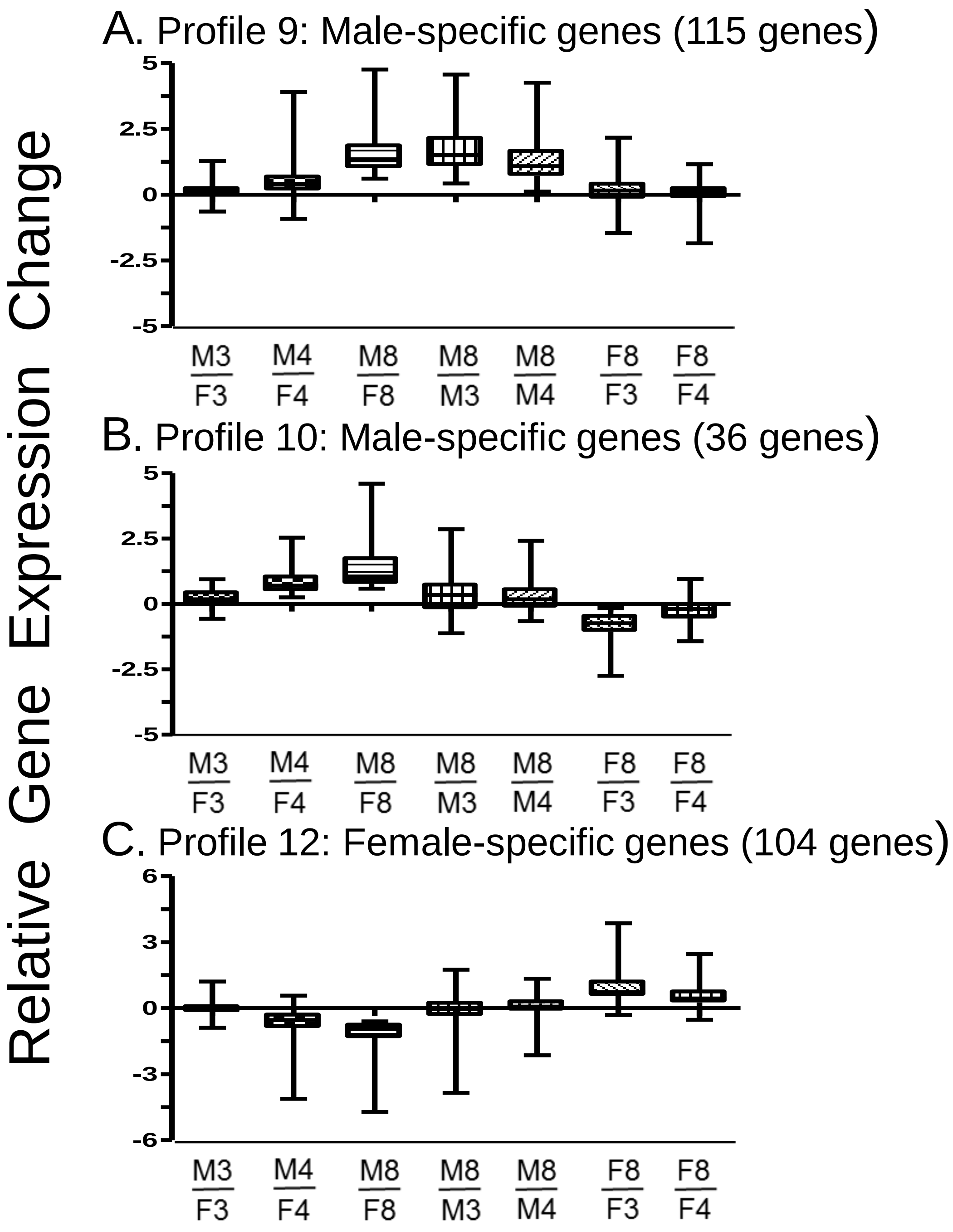

Additional File 4B.

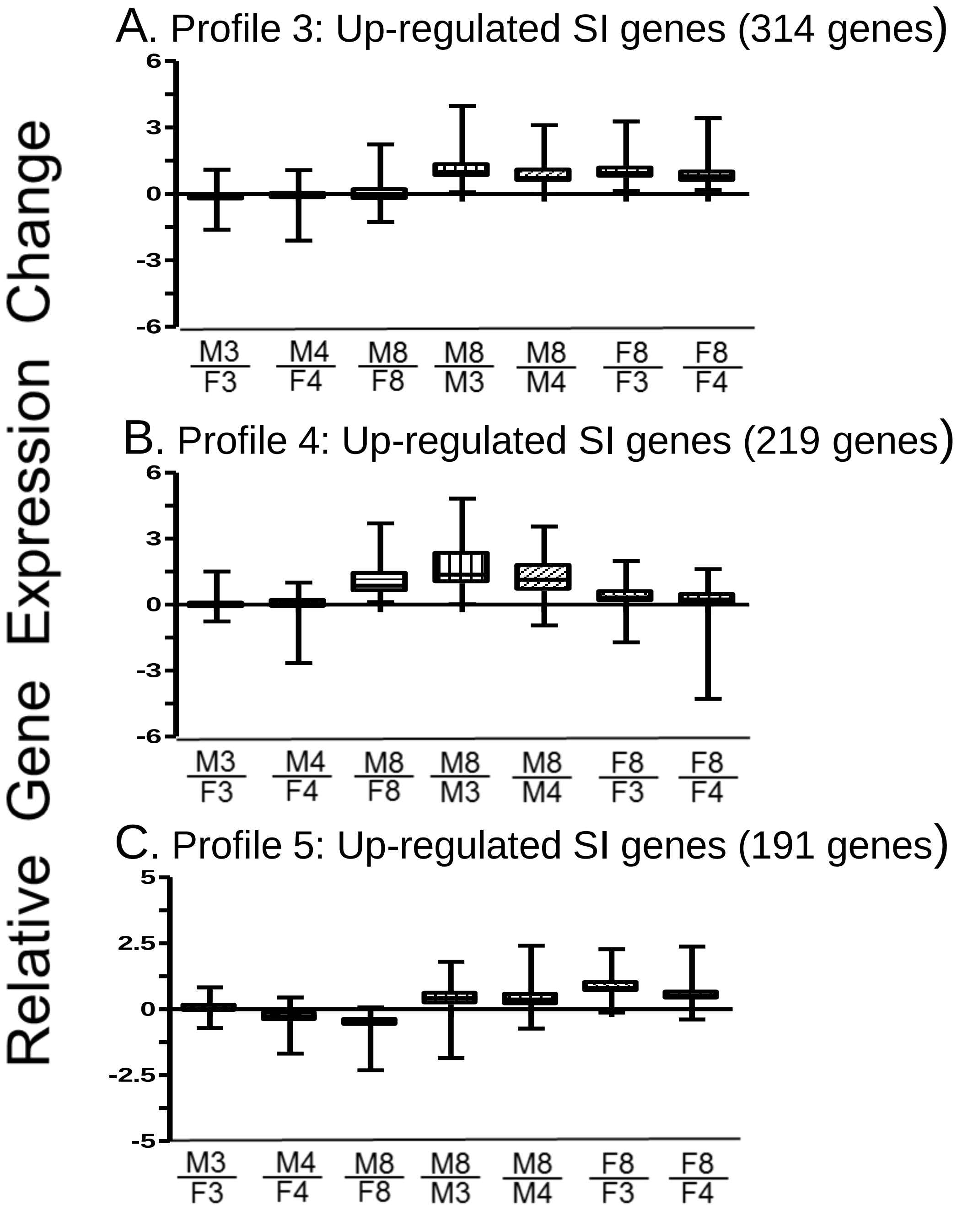

Additional File 4C.

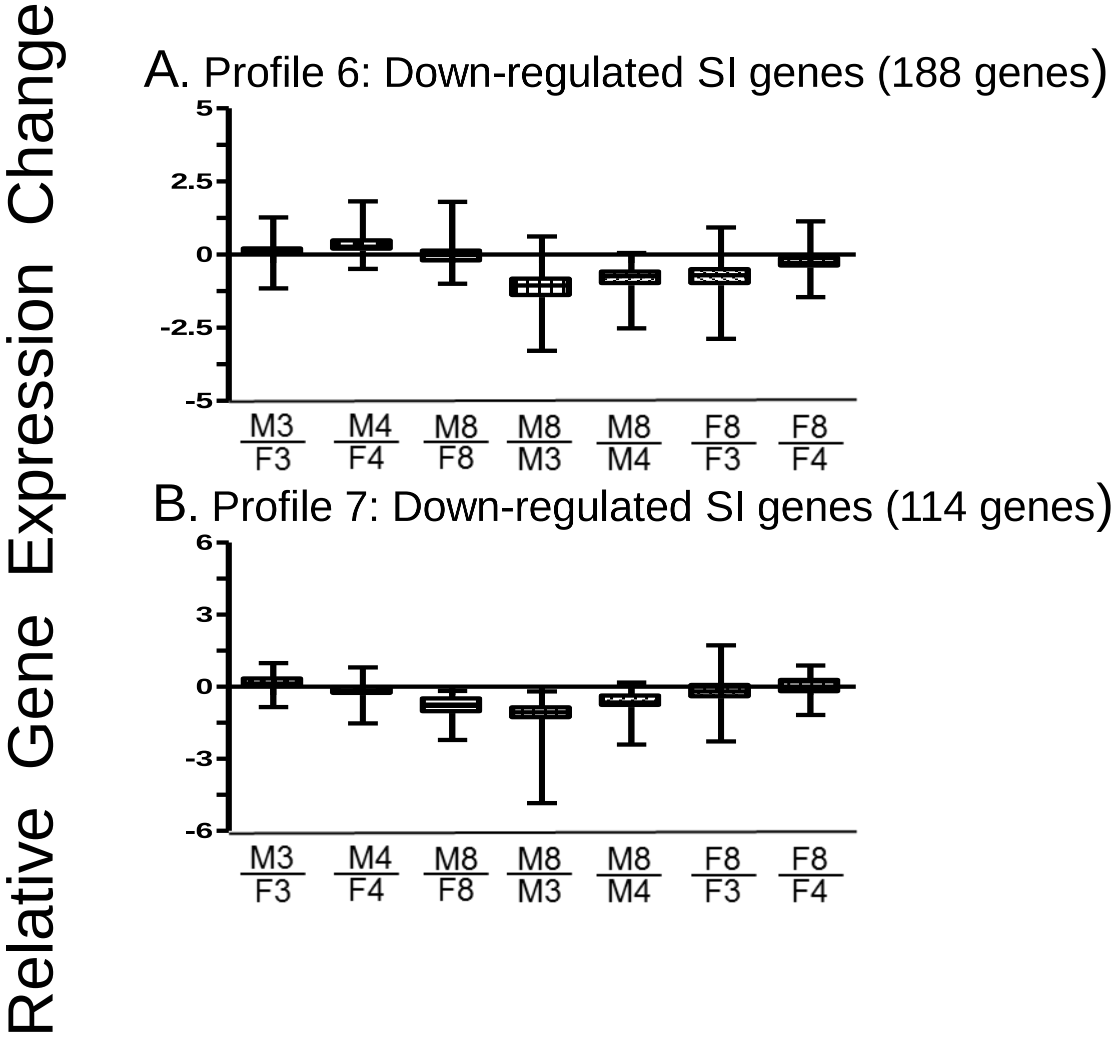

Supplement: Additional file 4 — A. Box and whisker plots representing gene expression patterns in STEM cluster profiles 9, 10, and 12. Boxes represent the 25th to the 75th percentile of gene expression ratios for each of the 7 microarray comparisons indicated below the X-axis. A horizontal line across each box indicates the median expression ratio. The whiskers that extend above and below each box represent the highest and the lowest values. M3, male at 3 wk; M4, male at 4 wk; M8, male at 8 wk; F3, female at 3 wk; F4, female at 4 wk; and F8, female at 8 wk. (A) Profile 9 is comprised of male-specific genes that are up regulated from 3 wk to 8 wk and from 4 wk to 8 wk in male liver but not in female liver. (B) Profile 10 is comprised of male-specific genes that are, on average, slightly up regulated from 3 wk to 8 wk and from 4 wk to 8 wk in male liver and slightly down regulated from 3 wk to 8 wk in female liver. (C) Profile 12 is comprised of female-specific genes that are up regulated from 3 wk to 8 wk and from 4 wk to 8 wk in female liver. B. Box and whisker plots representing gene expression patterns in STEM cluster profiles 3, 4, and 5. Graphs are presented as in Additional file 4A. (A) Profile 3 is comprised of sex-independent (SI) genes that are up regulated from 3 wk to 8 wk and from 4 wk to 8 wk in both male and female liver. (B) Profile 4 is comprised of sex-independent genes that are up regulated from 3 wk to 8 wk and from 4 wk to 8 wk in male liver. (C) Profile 5 is comprised of sex-independent genes that are up regulated from 3 wk to 8 wk and from 4 wk to 8 wk in female liver, but show weak up regulation in male liver. C. Box and whisker plots representing gene expression patterns in STEM cluster profiles 6 and 7. Graphs are presented as in Additional file 4A. (A) Profile 6 is comprised of sex-independent (SI) genes that are down regulated from 3 wk to 8 wk in both male and female liver and from 4 wk to 8 wk in male liver. (B) Profile 7 is comprised of sex-independent genes that are d [file 2042-6410-3-9-S4.PDF]
