# Supplementary figures and images for: Sex-specific mouse liver gene expression: genome-wide analysis of developmental changes from pre-pubertal period to young adulthood
Source: Biol Sex Differ. 2012 Apr 4;3:9. doi: 10.1186/2042-6410-3-9 (PMC3350426; doi:10.1186/2042-6410-3-9)

## Male-specific Genes (microarray intensities)

A. Hsd3b5 (TFS: 14.011-1000)

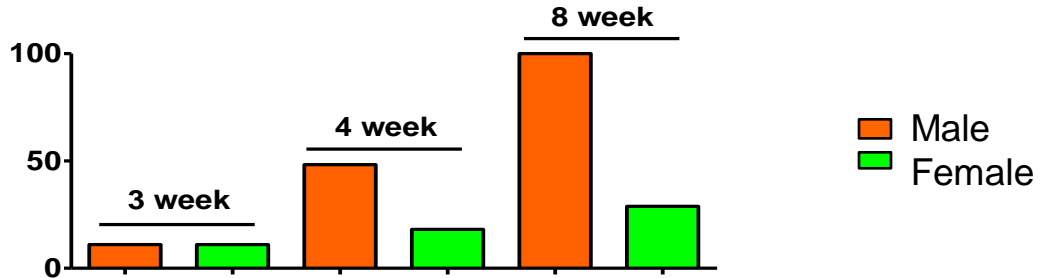

B. C6 (TFS: 30.011-1100)

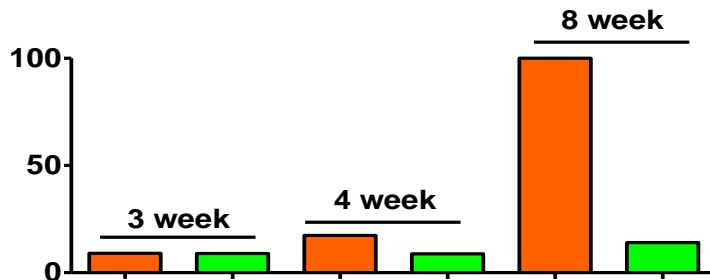

C. Cyp2u1 (TFS: 28.001-1100)

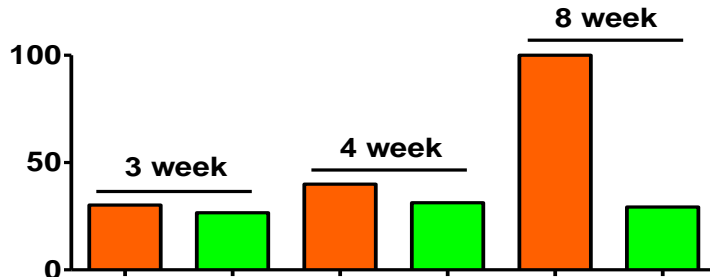

D. GSTpi (TFS: 60.001-1120)

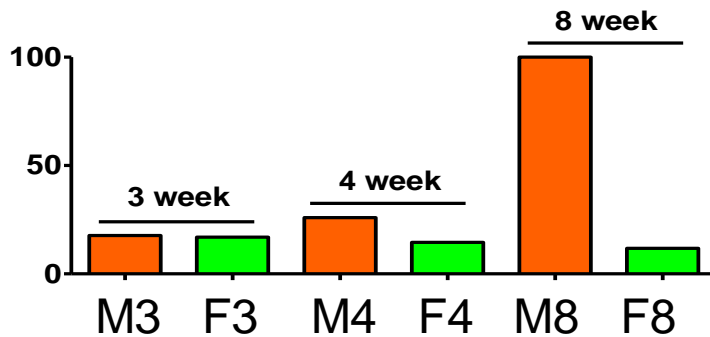

Supplement: Additional file 8 — Microarray intensities of male-specific genes. These graphs present the relative microarray intensities of each indicated male-specific gene. Expression patterns are very similar to those determined by qPCR analysis in Figure 4. [file 2042-6410-3-9-S8.PDF]

**Female-specific Genes (microarray intensities)**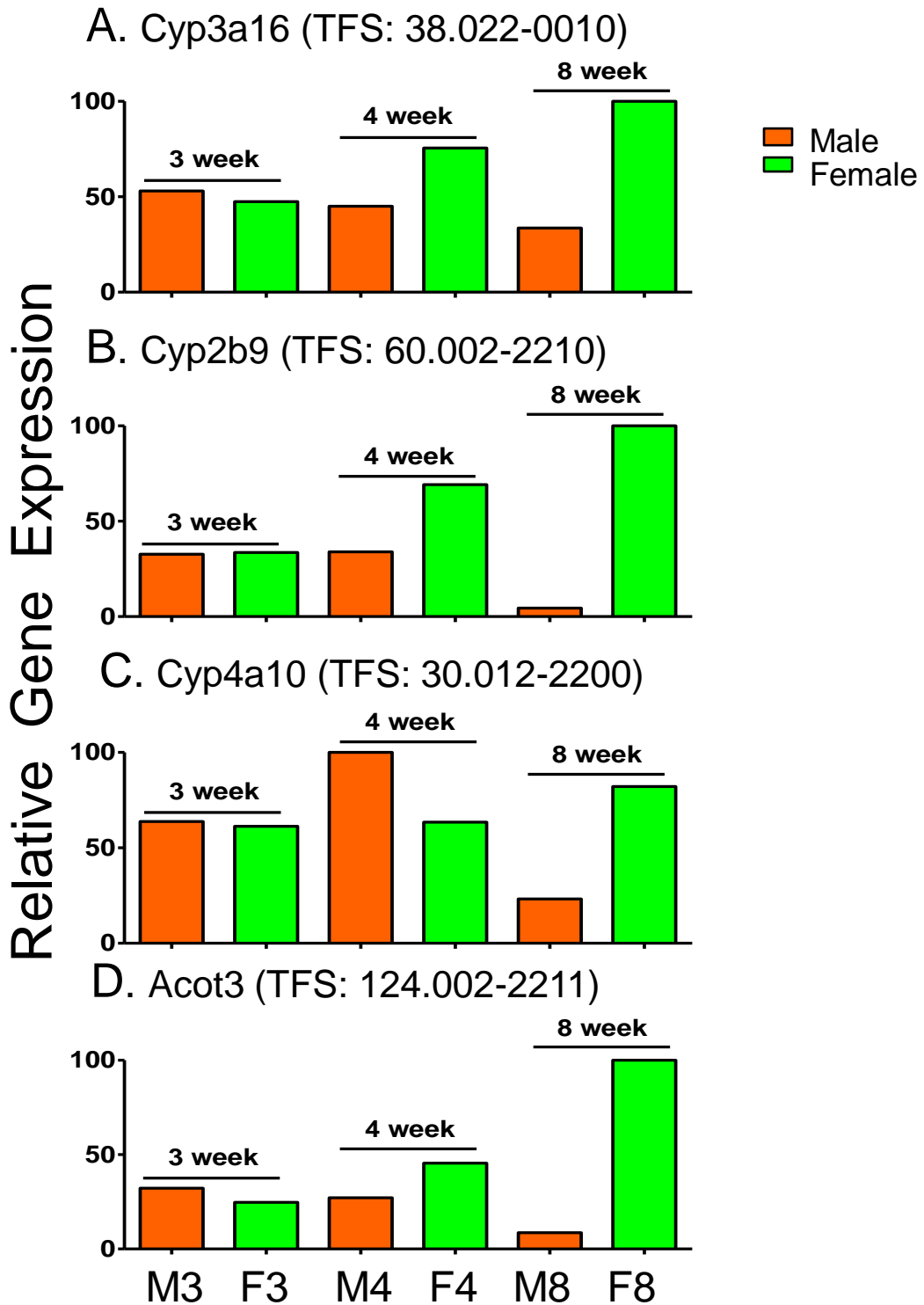

Supplement: Additional file 9 — Microarray intensities of female-specific genes. These graphs present the relative microarray intensities of each indicated female-specific gene. Expression patterns are very similar to those determined by qPCR analysis in Figure 5. The exception is Cyp3a16, whose microarray probe cross-hybridizes with Cyp3a11, a non-sex-specific gene, which results in a much lower sex-difference that that observed by qPCR. [file 2042-6410-3-9-S9.PDF]
